# Supplementary material for: Structure and Biosynthetic Gene Cluster of Sulfated Capsular Polysaccharide from the Marine Bacterium Vibrio sp. KMM 8419
Source: Int J Mol Sci. 2024 Dec 1;25(23):12927. doi: 10.3390/ijms252312927 (PMC11641403; doi:10.3390/ijms252312927)
Supplement: Supplementary file 1 [file ijms-25-12927-s001.zip › Supplementary_CPS_Vibrio CB1-14.pdf]

Structure and Biosynthetic Gene Cluster of Sulfated Capsular Polysaccharide from the Marine Bacterium *Vibrio* sp. KMM 8419

Maxim S. Kokoulin [1,*], Yulia V. Savicheva [1], Nadezhda Y. Otstavnykh [1], Valeria V. Kurilenko [1], Dmitry A. Meleshko [2] and Marina P. Isaeva [1,*]

[1] G.B. Elyakov Pacific Institute of Bioorganic Chemistry, Far Eastern Branch, Russian Academy of Sciences, 159/2, Prospect 100 let Vladivostoku, Vladivostok 690022, Russia; iu.savicheva0@yandex.ru (Y.V.S.); chernysheva.nadezhda@gmail.com (N.Y.O.); valerie@piboc.dvo.ru (V.V.K.)

[2] Principal Engineering School, ITMO University, 9, Lomonosova Street, St. Petersburg 191002, Russia; meleshko.dmitrii@gmail.com

* Correspondence: maxchem@mail.ru (M.S.K.); issaeva@gmail.com (M.P.I.)

Supplementary Materials

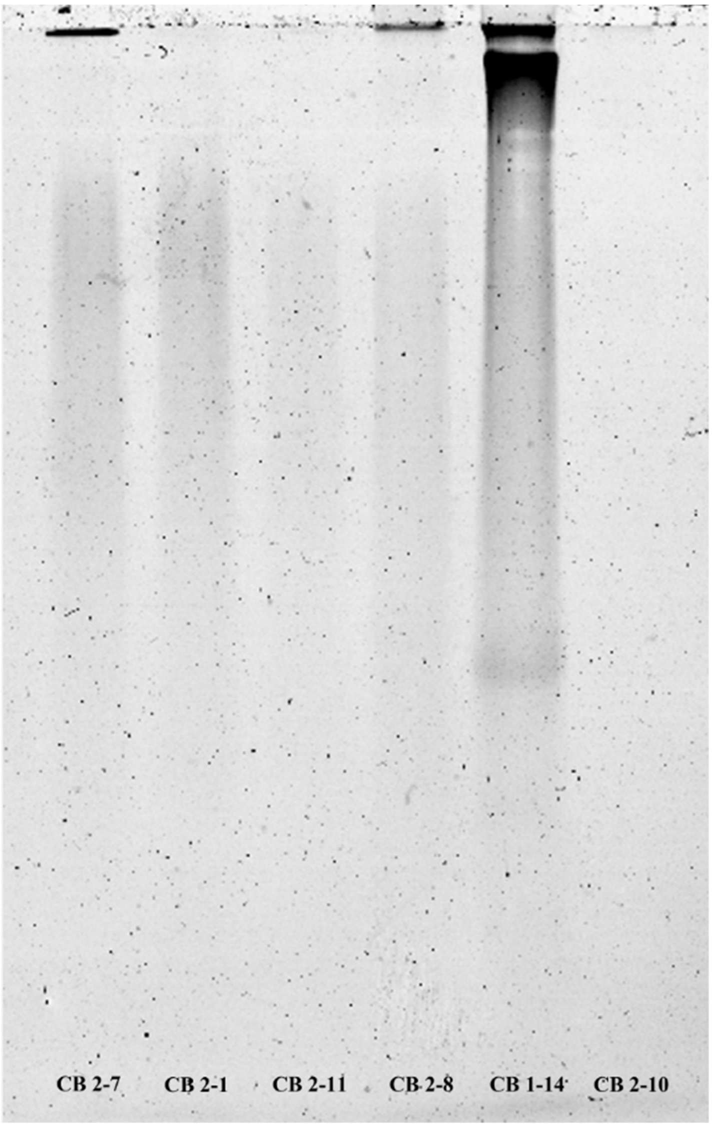

Figure S1. Toluidine blue-stained electrophoresis of sulfated polysaccharides from *Vibrio* spp. in a polyacrylamide gel.

Table S1. List of genes of a 95-kb locus *Vibrio* sp. KMM 8419 involved in biosynthesis of the sulfated capsular polysaccharide

ORF	Genomic Locus*	Gene name	Length bp (aa)	Product name, EC	Family and domain databases
ORF_343	PG915_01835	*yjbE*	446 (74)	Exopolysaccharide production protein YjbE	No GO Terms
ORF_344	PG915_01840	*yjbF*	618 (205)	YjbF family lipoprotein/ Putative lipoprotein GfcB	*IPR021308* Group 4 capsule polysaccharide formation lipoprotein GfcB;
ORF_345	PG915_01845	*yjbG*	810 (269)	Capsule biosynthesis GfcC family protein	*IPR046459* Capsule biosynthesis GfcC-like, N-terminal; *IPR010425* Capsule biosynthesis GfcC-like, C-terminal
ORF_346	PG915_01850	*yjbH*	2196 (731)	Bacterial lipoprotein (DUF940) YjbH domain-containing protein	*IPR010344* Exopolysaccharide biosynthesis protein YbjH
ORF_347	PG915_01855	*wecA*	1068 (355)	Undecaprenyl-phosphate alpha-N-acetylglucosaminyl 1-phosphate transferase, EC:2.7.8.33	*IPR012750* ECA_WecA-rel; *IPR000715* Glycosyl_transferase_4
ORF_348	PG915_01860	*cysC*	636 (211)	Adenylyl-sulfate kinase, EC:2.7.1.25	IPR002891
ORF_349	PG915_01865	sulfate permease	1713 (570)	SLC13 family permease	*IPR006037* Regulator of K+ conductance, C-terminal; *IPR004680* Citrate transporter-like domain;
ORF_350	PG915_01870	*cysN*	1407 (468)	Sulfate adenylyltransferase subunit 1, EC:2.7.7.4	*IPR011779* Sulphate adenylyltransferase, large subunit
ORF_351	PG915_01875	-	354 (117)	Four helix bundle protein	*IPR012657* 23S rRNA-intervening sequence protein TIGR02436 four helix bundle protein
ORF_352	PG915_01880	*cysD*	909 (302)	Sulfate adenylyltransferase subunit 2, EC:2.7.7.4	*IPR011784* Sulphate adenylyltransferase, small subunit
ORF_353	PG915_01885	*kpsT*	651 (216)	Polysaccharide/polyol phosphate ABC transporter ATP-binding protein	*IPR003439* ABC transporter-like, ATP-binding domain IPR003593 AAA+ ATPase domain *IPR015860* ABC transporter, teichoic acids export TagH-like
ORF_354	PG915_01890	-	1164 (387)	Sulfotransferase domain-containing protein	No GO terms
ORF_355	PG915_01895	GT61 (DUF563)	1170 (389)	Glycosyltransferase family 61 protein	*IPR049625* Glycosyltransferase 61, catalytic domain
ORF_356	PG915_01900	P-loop NTPase	858 (285)	Sulfotransferase domain-containing protein	*IPR027417* P-loop containing nucleoside triphosphate hydrolase
ORF_357	PG915_01905	*rgpF*	2034 (677)	HAD-IA family hydrolase; Rhamnan synthesis protein F	*IPR007739* Rhamnan synthesis F *IPR036412* HAD-like superfamily
ORF_358	PG915_01910	-	1041 (346)	HAD family hydrolase	No GO terms

ORF_359	PG915_01915	RCC1	4569 (1522)	RCC1 repeat-containing protein	*IPR028974* TSP type-3 repeat<br>*IPR009091* Regulator of chromosome condensation 1/beta-lactamase-inhibitor protein II
ORF_360	PG915_01920	GT2	1728 (575)	Glycosyltransferase 2-like domain-containing protein	*IPR001173* Glycosyltransferase 2-like
ORF_361	PG915_01925	GT4	1230 (409)	Glycosyl transferase family 1	*IPR001296* Glycosyl transferase, family 1<br>*IPR028098* Glycosyltransferase subfamily 4-like, N-terminal domain
ORF_362	PG915_01930	sulfotransferase	1140 (379)	Sulfotransferase domain-containing protein	*IPR027417* P-loop containing nucleoside triphosphate hydrolase<br>*PF13469* Sulfotransferase family
ORF_363	PG915_01935	P-loop NTPase	999 (332)	Sulfotransferase domain-containing protein	*IPR027417* P-loop containing nucleoside triphosphate hydrolase
ORF_364	PG915_01940	GT61 (DUF563)	975 (324)	Capsular biosynthesis protein	*IPR049625* Glycosyltransferase 61, catalytic domain
ORF_365	PG915_01945	-	1908 (635)	Uncharacterized protein	No GO Terms
ORF_366	PG915_01950	-	411 (136)	Glycosyltransferase	No GO Terms
ORF_367	PG915_01955	-	1386 (461)	Right handed beta helix domain-containing protein	*IPR012334* Pectin lyase fold<br>*G3DSA:2.160.20.10* Single-stranded right-handed beta-helix, Pectin lyase-like
ORF_368	PG915_01960	integrin	2400 (799)	Protective antigen Ca-binding domain-containing protein	*IPR028994* Integrin alpha, N-terminal<br>*PTHR37467* EXPORTED CALCIUM-BINDING GLYCOPROTEIN-RELATED
ORF_369	PG915_01965	-	1230 (409)	ATPase	No GO Terms
ORF_370	PG915_01970	-	1380 (459)		*IPR012334* Pectin lyase fold<br>*IPR011050* Pectin lyase fold/virulence factor
ORF_371	PG915_01975	-	2919 (1871)	Calcium-binding acidic-repeat protein	*IPR028994* Integrin alpha, N-terminal<br>*PTHR37467* EXPORTED CALCIUM-BINDING GLYCOPROTEIN-RELATED
ORF_372	PG915_01980	-	7197 (2398)	Subtilisin-like serine protease	*IPR015500* Peptidase S8, subtilisin-related<br>*IPR002102* Cellulosome anchoring protein, cohesin domain<br>*IPR002909* IPT domain<br>*IPR003961* Fibronectin type III
ORF_373	PG915_01985	integrin	5130 (1706)		*IPR028994* Integrin alpha, N-terminal<br>*IPR028974* TSP type-3 repeat
ORF_374	PG915_01990	*galF*	903 (300)	UTP--glucose-1-phosphate uridylyltransferase EC:2.7.7.9	*IPR005771* UTP--glucose-1-phosphate uridylyltransferase, bacterial/archaeal-type

ORF_375	PG915_01995	transposase	402 (133)	Transposase DDE domain protein	*IPR047647* ISAs1 transposase
ORF_376	PG915_02000	*dnaC*	468 (155)	IstB domain protein ATP-binding protein DNA replication protein DnaC	*PF01695* IstB-like ATP binding protein *IPR027417* P-loop containing nucleoside triphosphate hydrolase
ORF_377	PG915_02005	*dnaC*	336 (111)	DNA replication protein DnaC	*PF01695* IstB-like ATP binding protein
ORF_378	PG915_02010	integrase	999 (332)	Integrase core domain protein	*IPR001584* Integrase, catalytic core *IPR012337* Ribonuclease H-like superfamily *IPR017894* HTH domain, IS21 transposase-type
ORF_379	PG915_02015	transposase	810 (269)	ISAs1 family transposase	*IPR051698* Transposase 11-like *IPR047647* ISAs1 transposase *IPR032806* H repeat-associated protein, N-terminal
ORF_380	PG915_02020	transposase	1128 (375)	Transposase DDE domain protein	*IPR051698* Transposase 11-like *IPR047647* ISAs1 transposase *IPR032806* H repeat-associated protein, N-terminal
ORF_381	PG915_02025	*bcbE*-like (GT2)	726 (241)	Capsular biosynthesis protein	*IPR016873* Capsular polysaccharide biosynthesis protein, BcbE, predicted *IPR029044* Nucleotide-diphospho-sugar transferases
ORF_382	PG915_02030	APH	1581 (526)	Capsular polysaccharide biosynthesis protein	*IPR002575* Aminoglycoside phosphotransferase *IPR011009* Protein kinase-like domain superfamily
ORF_383	PG915_02035	*ecbF*	399 (132)	Capsular biosynthesis protein	*IPR010039* Capsule biosynthesis phosphatase *IPR010033* HAD-superfamily phosphatase, subfamily IIIC
ORF_384	PG915_02040	-	1467 (488)	Tetratricopeptide repeat-containing protein	No GO terms
ORF_385	PG915_02045	-	1257 (418)	Alpha/beta hydrolase	*IPR029058* Alpha/Beta hydrolase fold *IPR011990* Tetratricopeptide-like helical domain superfamily
ORF_386	PG915_02050	*sft1*	843 (280)	Sulfotransferase domain-containing protein	*IPR037359* Heparan sulfate sulfotransferase *IPR027417* P-loop containing nucleoside triphosphate hydrolase
ORF_387	PG915_02055	*rfaB* (GT4)	3321 (1106)	Glycosyl transferase family 1_4 protein	*IPR028098* Glycosyltransferase subfamily 4-like, N-terminal domain *IPR001296* Glycosyl transferase, family 1 Sulfoquinovosyl transferase
ORF_388	PG915_02060	*rmlA*	897 (298)	Glucose-1-phosphate thymidylyltransferase	*IPR005907* Glucose-1-phosphate thymidylyltransferase, short form *IPR005835* Nucleotidyl transferase domain

ORF_389	PG915_02065	*rmlD*	891 (296)	dTDP-4-dehydrorhamnose reductase	*IPR005913* dTDP-4-dehydrorhamnose reductase family *IPR029903* RmlD-like substrate binding domain
ORF_390	PG915_02070	*rfbF*-like	930 (309)	Glycosyltransferase family 2 protein	*IPR029044* Nucleotide-diphospho-sugar transferases *PTHR43179* RHAMNOSYLTRANSFERASE WBBL
ORF_391	PG915_02075	*wbbL*	831 (276)	Glycosyltransferase family 2 protein	*IPR029044* Nucleotide-diphospho-sugar transferases *PTHR43179* RHAMNOSYLTRANSFERASE WBBL
ORF_392	PG915_02080	*rmlC*	546 (181)	dTDP-4-dehydrorhamnose 3,5-epimerase	*IPR000888* dTDP-4-dehydrorhamnose 3,5-epimerase-like
ORF_393	PG915_02085	*kpsE*	1461 (486)	Lipopolysaccharide biosynthesis protein	*PTHR32309* TYROSINE-PROTEIN KINASE
ORF_394	PG915_02090	*kpsM*	786 (261)	Transport permease protein	*IPR047817* ABC-2 type transporter, transmembrane domain, bacterial-type
ORF_395	PG915_02095	*kpsD*	1806 (601)	Polysaccharide export protein	*IPR049712* Polysaccharide export protein
ORF_396	PG915_02100	-	366 (121)	Lipoprotein	No GO Terms
ORF_397	PG915_02105	DHH	966 (321)	Acetyltransferase	*IPR038763* DHH phosphoesterase superfamily
ORF_398	PG915_02110	*rmlB*	1062 (353)	dTDP-glucose 4,6-dehydratase	*IPR005888* dTDP-glucose 4,6-dehydratase *IPR016040* NAD(P)-binding domain
ORF_399	-	-	129 (42)	(no results found)	No GO Terms
ORF_400	PG915_02115	-	339 (112)	Alpha/beta hydrolase	No GO Terms
ORF_401	PG915_02120	*rig*	1350 (449)	Metallo-beta-lactamase family protein RNA-specific	*IPR001279* Metallo-beta-lactamase *IPR022712* Beta-Casp domain *IPR011108* Zn-dependent metallo-hydrolase, RNA specificity domain
ORF_402	PG915_02125	*wzx*	1275 (424)	Flippase	*IPR002797* Polysaccharide biosynthesis protein *IPR029303* Polysaccharide biosynthesis protein, C-terminal domain
ORF_403	PG915_02130	*ctgA*	810 (269)	Glycosyl transferase family 2	*IPR010446* Beta-1,4-N-acetylgalactosaminyltransferase *PF06306* Beta-1,4-N-acetylgalactosaminyltransferase (CgtA)
ORF_404	PG915_02135	sulfotransferase	672 (223)	Sulfotransferase family protein	*IPR018011* Carbohydrate sulfotransferase 8-10 *IPR005331* Sulfotransferase
ORF_405	PG915_02140	*kpsS*	1167 (388)	Capsule polysaccharide biosynthesis protein	*IPR007833* Capsule polysaccharide biosynthesis
ORF_406	PG915_02145	GT4	945 (314)	Glycosyltransferase	*PF13692* Glycosyl transferases group 1
ORF_407	PG915_02150	*capM*-like (GT4)	1131 (376)	Glycosyltransferase involved in cell wall bisynthesis	*IPR028098* Glycosyltransferase subfamily 4-like, N-terminal domain *IPR001296* Glycosyl transferase, family 1

					PTHR45947 SULFOQUINOVOSYL TRANSFERASE SQD2<br>*cd03808* capsular polysaccharide biosynthesis glycosyltransferase CapM and similar proteins
ORF_408	PG915_02155	*gnu*	912 (303)	UDP-glucose 4-epimerase/UDP-N-acetyl-α-D-quinovosamine dehydrogenase	*IPR001509* NAD-dependent epimerase/dehydratase
ORF_409	PG915_02160	*epsL*	555 (184)	Lipid carrier--UDP-N-acetylgalactosaminyltransferase	*IPR003362* Bacterial sugar transferase<br>*PTHR30576* COLANIC BIOSYNTHESIS UDP-GLUCOSE LIPID CARRIER TRANSFERASE
ORF_410	PG915_02165	*pglF*	1914 (637)	UDP-N-acetylglucosamine 4,6-dehydratase	*IPR003869* Polysaccharide biosynthesis protein, CapD-like domain<br>*cd05237* UDP-Glcnac (UDP-linked N-acetylglucosamine) inverting 4,6-dehydratase, extended (e) SDRs<br>*IPR029063* S-adenosyl-L-methionine-dependent methyltransferase superfamily
ORF_411	PG915_02170	*ugd*	1167 (388)	UDP-glucose 6-dehydrogenase<br>for UDP-glucuronic acid (GlcA) biosynthesis	*IPR028357* UDP-glucose 6-dehydrogenase, bacterial type<br>*IPR017476* UDP-glucose/GDP-mannose dehydrogenase

* The GenBank accession number for chromosomes 1 is CP115920.1

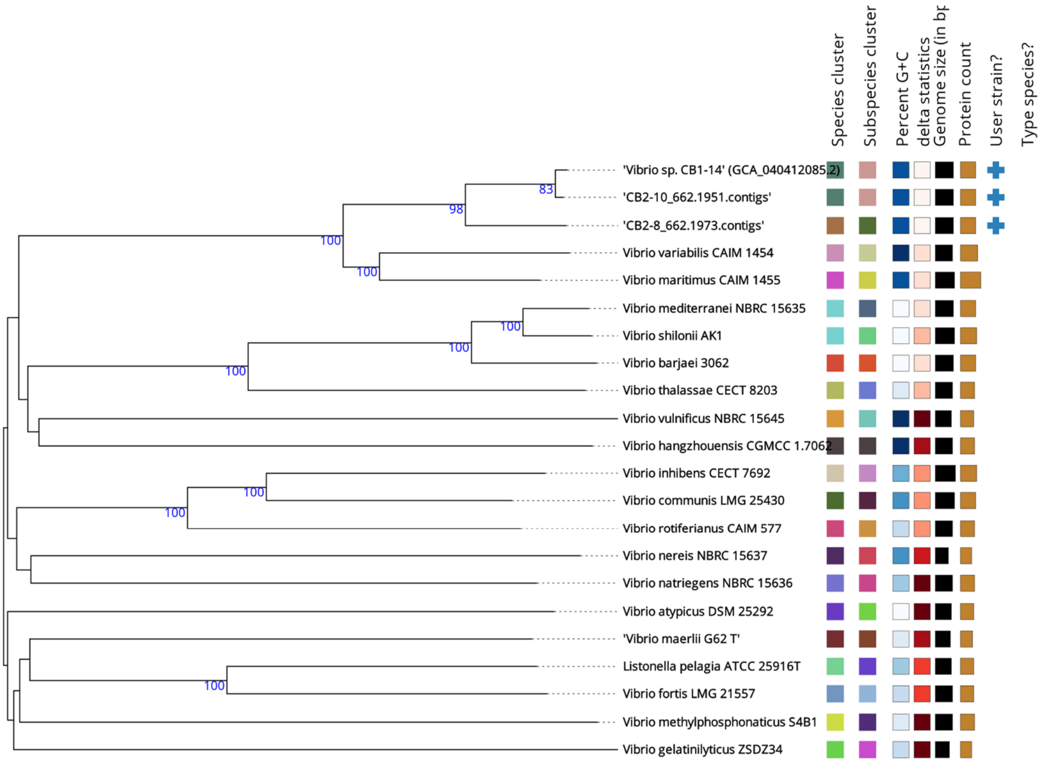

Figure S2. ML genomic tree showing phylogenetic relationships of the strains KMM 8419, KMM 8420 and KMM 8434 and members of the genus *Vibrio*. Bootstrap values shown as percentage greater than 75.
